# Health care professionals’ perceptions towards lifelong learning in palliative care for general practitioners: a focus group study

**DOI:** 10.1186/1471-2296-15-36

**Published:** 2014-02-19

**Authors:** Peter Pype, Linda Symons, Johan Wens, Bart Van den Eynden, Ann Stes, Myriam Deveugele

**Affiliations:** 1 Department of Family Medicine and Primary Health Care, Ghent University, UZ-6 K3, De Pintelaan 185, 9000 Gent, Belgium; 2 Department of Primary and Interdisciplinary Care Antwerp - PICA, University of Antwerp, Antwerp, Belgium; 3 Institute for Education and Information Sciences, University of Antwerp, Antwerp, Belgium

**Keywords:** Interprofessional learning, Workplace learning, Interprofessional collaboration, Primary care, Continuing professional development

## Abstract

**Background:**

There is a growing need for palliative care. The majority of palliative patients prefer their general practitioner (GP) to organize their palliative home care. General practitioners need a range of competences to perform this task. However, there has been no general description so far of how GPs keep these competences up-to-date. The present study explores current experiences, views and preferences towards training and education in palliative care among GPs, palliative home-care professionals and professionals from organizations who provide training and education.

**Methods:**

Five focus groups were brought together in Belgium, with a total of 29 participants, including members of the three categories mentioned above. They were analysed using a constant comparison method.

**Results:**

The analysis revealed that undergraduate education and continuing medical education (CME) while in practice, is insufficient to prepare GPs for their palliative work. Workplace learning (WPL) through collaboration with specialized palliative home-care nurses seems to be a valuable alternative.

**Conclusions:**

The effectiveness of undergraduate education might be enhanced by adding practical experience. Providers of continuing medical education should look to organize interactive, practice-based and interprofessional sessions. Therefore, teachers need to be trained to run small group discussions. In order to optimize workplace learning, health care professionals should be trained to monitor each other’s practice and to provide effective feedback. Further research is needed to clarify which aspects of interprofessional teamwork (e.g. professional hierarchy, agreements on tasks and responsibilities) influence the effectiveness of workplace learning.

## Background

Over the last few decades there has been an increase worldwide in the number of patients suffering from advanced cancer and severe non-malignant diseases. The majority of these palliative patients prefer to spend their final days at home being cared for by their general practitioner (GP) rather than in a hospice or hospital setting [1-6]. In general, GPs accept this task as an important part of their lifelong commitment to patients [7]. To be able to deliver high-quality care, GPs need a specific set of palliative-care competences. The European Association for Palliative Care (EAPC) has listed these competences in their undergraduate and postgraduate curriculum suggestions [8,9]. Medical schools can make use of these suggestions to implement palliative-care education in medical curricula. They can also be used as an information source for providers of continuing medical education (CME). Within the framework of this study, we made use of the word ‘CME’ to appoint the officially organized educational sessions required for the periodic recertification of doctors.

In many countries there is no comprehensive undergraduate or postgraduate palliative-care curriculum for medical students [10]. In EAPC’s recent ‘Atlas of Palliative Care in Europe’, the development of palliative care in 30 European countries is described. Among these 30 countries, 13 are reported to have some palliative-care teaching in all medical schools, while for 15 countries it is reported that in part of the medical schools there is some teaching (0-64% of medical schools). For 2 countries nothing is reported on it at all [10]. Therefore, it is not known how GPs acquire the necessary competences to deliver high-quality palliative care. One might think that practical experience leads to a higher level of competence but the literature suggests that the quality of care provided (measured by the adherence to guidelines as a proxy indicator) is inversely proportional to the number of years in practice [11,12]. Moreover, GPs only have a small number of palliative patients a year so the occasions to gain experience are limited [13,14]. This lack of experience and the associated lack of self-confidence is internationally recognized [7]. Nevertheless, it is worthwhile to consider learning through experience in more detail.

In many countries, GPs collaborate with community-based palliative home-care teams (PHCTs) to provide effective palliative home care [10,15,16].

The literature on workplace learning (WPL) acknowledges that working and learning are inseparable and fundamental [17,18]. Interprofessional learning is ‘learning arising from the interaction between members of two or more professions’ and may happen spontaneously, in an implicit way, when health-care providers from different disciplines work together in taking care of the same patient [18,19]. In our case, we expect GPs to learn through collaboration with the more experienced PHCT nurses.

The two ways of learning we have mentioned (classroom-based learning and workplace learning) draw on different learning perspectives [20]. Whereas classroom-based learning is primarily intended for knowledge transmission (even though some formats such as workshops, small-group discussions and role play incorporate the putting into practice of theory), workplace learning occurs through actively engaging in the activities of the workplace [17,21,22]. The interaction between the individuals and the environment thereby offers situated learning opportunities where new knowledge is co-constructed. To be able to gain the most comprehensive insight into GPs’ preferences for acquiring palliative-care competences, we sought the views and ideas of all the different parties involved in GPs’ palliative-care learning: GPs, CME providers and PHCT members. Therefore we conducted focus groups with GPs only and focus groups comprising both PHCT members and CME providers. The research questions of this study were:

•What are the current experiences of GPs, CME providers and PHCT members with palliative-care education for GPs?

•What are the views on and preferences for future palliative-care education for GPs according to GPs, CME providers and PHCT members respectively?

## Methods

### Setting

In Belgium, GPs have a central position in primary care. They deliver medical care and coordinate the involvement of other health-care professionals e.g. community nurses. Near the end of a patient’s life, during palliative home care, GPs remain responsible. With respect to education, both on the undergraduate and the postgraduate levels, medical schools offer some palliative-care curriculum items but there are no official recommendations as to content or didactics. Most undergraduate palliative-care education is classroom-based. During traineeships, GP trainees gather clinical experience under the supervision of an experienced GP, but no official requirements are provided. A recent survey of continuing medical education (CME) in palliative care has uncovered large content gaps, an under-usage of appropriate educational techniques and an absence of evaluation of the impact of CME on clinical practice [23].

Palliative-care services are well-developed in both home care and hospital care. Palliative home-care teams cover the entire country, and every GP can have recourse to a PHCT when needed. These PHCTs comprise specialized palliative-care nurses, physicians with specialist training in palliative care, and psychologists. The nurses generally undertake the majority of home visits, and in doing so support the GPs in their job. The PHCT physicians and psychologists mainly advise and support the nurses during team meetings.

### Design

A qualitative design using focus group discussions was chosen because the interaction between participants was expected to elicit the richest thoughts and ideas in an area where knowledge is scarce. Our main goal was to gather the GPs’ views and preferences on maintaining competences and not merely facts on how they maintained them.

### Recruitment and selection

In Belgium, each GP belongs to a peer review group which must meet four times a year as part of the recertification process. Two peer groups of GPs (one urban, one rural) were invited to participate in this research (n = 12) as a convenient sample choice near the hometown of two researchers. The main topics explored in these focus groups were: the acquisition of basic palliative-care competences, maintaining competences and collaboration with other professionals. The topics are listed in Table 1.

**Table 1:** Topics discussed in the focus groups

**Topics discussed in the focus groups with GPs**	**Topics discussed in the focus groups with PHCTs and CME providers**
Acquisition of basic palliative-care competences	Interactions between participants of the focus groups and GPs
*(Where did you get your basic knowledge and competences?; How did you handle your first palliative patients? What did you miss during your education?)*	*(How do you come into contact with GPs?; What are the questions they put to you?; Which needs do GPs formulate to you? Are these the same needs that you see as collaborating professionals?)*
Maintaining competences	Continuing medical education
*(Where and how have you been learning about palliative care?; Which kind of learning do you prefer and why?; How does the learning influence your practice as a GP?)*	*(How do you organize CME?; How do you take into account the needs and preferences of GPs with respect to CME? What are your preferences with respect to CME?)*
Collaboration with other professionals.	Collaboration with GPs
*(With whom do you collaborate in palliative care?; Which contacts with other professionals have been most educational?; How does this influence your practice as a GP?)*	(*What is your perception of the collaboration with GPs?; Are GPs communicating to you about the collaboration?; Are you instructing GPs on interdisciplinary collaboration?)*

In addition, members of PHCTs and providers of CME were contacted. Mail surveys were sent out to every CME provider in Flanders as part of a larger study, and they were asked whether they would agree to participate in a focus group study [23]. Since PHCTs also provide education for GPs, they were included in this mailing. These focus groups explored the following issues: interactions between participants of the focus groups and GPs; continuing medical education and collaboration with GPs. The topics are listed in Table 1.

### Data generation

All focus group meetings lasted for approximately two hours. The participants gave their informed consent and were assured of their confidential participation and of the anonymization of any published quotes. Apart from the participants, other people taking part in the discussions were the facilitator (LS) and a clinical researcher (PP), who made field notes. All discussions were audio-taped and transcribed verbatim.

### Data analysis

An inductive approach was used to analyse the data, making use of a “constant comparison” method and its related open and axial coding techniques in which the emerging concepts are firmly grounded in the collected data [24]. Using open coding, two researchers (PP and LG) independently analysed a first transcript. Both researchers chose their codes independently. Afterwards, the codes were compared and discussed until a consensus was reached. After this, the next transcript was analysed and discussed in the same way. Using the resulting coding scheme, the first transcript was then reviewed again to check the validity of the codes. This was done by comparing codes and themes within and between transcripts. In this iterative way, all transcripts were analysed and discussed until a final set of themes was obtained. This final set of themes was presented for discussion to the other co-authors of this paper. The analysis was done using NVivo 8 software.

### Ethical approval

Ethical approval was granted by the ethical committee of the Gasthuiszusters Antwerp Hospital, Belgium.

## Results

In total, five focus group discussions were held with a total of 29 participants, all based in the Dutch-speaking part of Belgium.

Firstly, two focus groups were convened with seven and five GPs in each group. All invited GPs of the two peer review groups agreed to participate and were present.

Secondly, three focus groups were convened with PHCT members and CME providers (with six, six and five participants, respectively). The analysis of the last focus group transcript did not reveal any new themes but additional insights into the existing set of themes emerged.

The characteristics of the participants are shown in Table 2.

**Table 2:** Characteristics of the participants of the focus groups

**Focus group**	**Participant**	**Gender**	**Age**	**Profession**	Years in practice*	Practice setting**
Nr 1 (GPs)	1	Male	48	GP	21	Solo
	2	Male	59	GP	34	Solo
	3	Male	45	GP	18	Solo
	4	Male	51	GP	24	Solo
	5	Male	36	GP	9	Solo
	6	Male	33	GP	6	Solo
	7	Male	56	GP	30	Solo
Nr 2 (GPs)	1	Female	33	GP	6	Duo
	2	Female	47	GP	19	Duo
	3	Male	59	GP	33	Duo
	4	Male	43	GP	16	Duo
	5	Male	45	GP	20	Group
Nr 3 (PHCTs and CME providers)	1	Male	51	GP	20/11	CME/PAL
	2	Male	60	GP	14	PAL
	3	Female	43	Nurse	12	PAL
	4	Female	43	Nurse	1	CME
	5	Male	47	Nurse	8	PAL
	6	Male	54	GP	30/14	CME/PAL
Nr 4 (PHCTs and CME providers)	1	Male	40	GP	4	CME
	2	Male	50	GP	7	PAL
	3	Male	37	Neurologist	2	PAL
	4	Male	54	Psychologist	11	PAL
	5	Female	33	Nurse	11	PAL
	6	Male	52	GP	3	PAL
Nr 5 (PHCTs and CME providers)	1	Female	46	GP	2	PAL
	2	Female	46	Master in medical-social sciences	8	CME
	3	Male	36	Palliative-care physician	5	PAL
	4	Male	44	Geriatrician	5	PAL
	5	Male	53	Anaesthetist	24	PAL

*years in practice: for GP groups = years of GP practice; for PHCT and CME providers groups = years working in this organization.

**practice setting: for GP groups = solo, duo or group practice; for PHCT and CME providers groups = PHCT (palliative home-care team), CME (providers of continuing medical education), PAL (palliative-care organization).

Quotes have been provided on the basis of their being representative of the wider data and are labelled using the number of the focus group and the number of the participant within the group (e.g. FG1, P2). The quotes were translated from Dutch into English. The accuracy of the translations was verified by discussing the meaning of the quotes with one or more of the authors. The three participating groups shared opinions on many themes. Differing opinions will be highlighted.

### What are the current experiences of GPs, CME providers and PHCT members with palliative-care education for GPs?

a) Insufficiently prepared on graduation

Similarly to primary care in general, palliative care is considered as total care that is patient-centred and relationship-based. Consequently, GPs were willing to invest time and energy in delivering palliative care as they regarded this as being a full aspect of their job. Therefore, they need a certain set of palliative care competences, the acquisition of which should be initiated as part of the undergraduate curriculum. GPs stated unanimously that the undergraduate palliative-care curriculum was insufficient for acquiring basic competences to start their medical practice in palliative care.

*‘It is still a leap into the unknown. You may have had ten hours of theory or twenty hours of theory, but sooner or later you’ll have to take the plunge and deal with it in practice.’* (FG1, P4)

Some GPs reported a deficiency in theoretical knowledge upon graduation e.g. with respect to pharmacology, because of an excess of attention and lectures on psychology and communication skills.

*‘Ultimately that’s the most important aspect I think. The wish of the patient is to be free of pain, to die as comfortable as possible. Therefore you need medication, not conversation. ’* (FG1, P6)

Others stressed the necessity to adopt a palliative-care attitude (shifting the focus ‘from cure to care’) to ensure good care and pointed out the lack of it in undergraduate training.

*‘At a given time, you have a point where you go beyond the usual framework of a diagnosis, a therapy, making somebody better. That logic – which is fed to us during our training – has to be left behind and you see: now I am just going to look at what makes a person comfortable. It is a completely different logic…’* (FG2, P2)

b) Task description and professional choice

Although participants unanimously agreed that palliative-care delivery is part of the GP’s job, task perception and the level of involvement clearly varied. All physicians wished to acquire basic palliative-care competences. Some GPs limited their involvement in palliative care because of its time-consuming and emotionally exhausting nature. Others deliberately confined themselves to patient care within their general primary-care competences and questioned the benefits of acquiring advanced competences since PHCTs and medical specialists are easily accessible for advice. As a result there was a spectrum ranging from GPs who performed palliative care ‘on their own’ to GPs handing over most of the tasks to others, especially PHCT nurses.

‘If you have had a patient for 20 or 30 years and he has to die, we are never going to be able to let him, we try to keep him alive for as long as possible, you should really have special doctors for that.’ (FG1, P5)

Consequently, not all GPs needed the same competences and this was reflected in their expectations towards the medical curriculum.

*‘Honestly, it doesn’t appeal to me … I think for example, if you want to know how a syringe driver works. You can call the PHCT for a syringe driver. You have to know that there is such a thing and what the indications are for its use. But all the practical aspects, I don’t need to know that, honestly, I really don’t need to know that.’* (FG2, P3)

The notion that not all GPs needed to have specialist palliative-care competences was confirmed in the focus groups of CME providers and PHCT members. They stated that skilled GPs can act as consultants for their colleagues.

*‘I used to be upset about that: we’re not reaching the ones we should be reaching. On the other hand it becomes more and more like a ‘dripping effect’. If we have a core group of 50, 60 GPs in a region who regularly attend courses, that will drip through to the others. You’ll notice other GPs turning to them… And colleagues knowing that… I think that’s a good way of circulating things.’* (FG3, P1)

c) Two distinct ways of lifelong learning

The participants agreed that GPs do not necessarily need to become palliative-care specialists but mostly require knowledge and skills to handle common actual patient-care needs. As the knowledge base of palliative care continuously changes, participants from all groups expressed the need for lifelong education and training, thereby distinguishing two ways of learning: formal educational sessions (CME) and learning by doing (workplace learning).

Most of the GPs were not enthusiastic about the CME sessions. Courses were often considered to be too theoretical and did not match their actual (on-the-spot) learning needs. CME providers, PHCTs and GP organisations all state that they often prepare courses collaboratively. This may enhance the effectiveness of the courses by emphasizing a focus on the GPs’ educational needs and preferences. This strategy may cover ‘general educational needs’ of a local group of GPs but is insufficient to address every individual GP’s learning needs.

Some education providers share these ideas and are very pessimistic about CME in general. They state that GPs have to ‘sense’ what good palliative care is all about and that it cannot be put into words or training.

*‘I don’t really believe in education. I don’t really believe in training. I don’t believe in that. I have spent lots of time lecturing GPs on pain and symptom control. But after you’ve finished, and one month later they have forgotten already… then I get this feeling: we can offer them hundreds of hours of training in palliative care, it won’t work. Experiencing this collaboration, that will make a click.’* (FG3, P1)

As mentioned earlier, as GPs are confronted with patient-care needs, their on-the-spot learning needs manifest themselves. These learning needs are to be resolved instantly, which cannot be done by scheduled CME sessions, distanced in time. A much better way to address these learning needs is through workplace learning.

*‘…and then you learn through trial and error. Of course. So you make mistakes. You… I remember patient cases, palliative cases, where I’ve been thinking ‘oops I really overlooked that. I really should have done this differently’. I’ve also dealt with people the wrong way. Learning through trial and error. I think that has been my principal teacher.’* (FG2, P2)

For GPs, learning by doing is the most natural way of learning, often with the help or ‘under the supervision’ of experienced nurses. As such, doctors expressed no reluctance or barriers towards asking for these nurses’ advice.

*‘OK, fortunately there is nursing at home, people who have been on the go for 20 years, who know the ropes, who push you to allow that, to try it at home, administering morphine, you know what I mean, all those things.’* (FG1, P7)

‘There has been resistance in the beginning, but as they experience that the palliative nurses also have the expertise and the ability, they (the nurses) are able to reach a good position to negotiate with those GPs.’ (FG3, P3)

Working together with PHCTs in a structured way accentuates different aspects of palliative care on top of the mere medical aspects, and this also creates learning moments for GPs. In such collaboration, GPs learn to shift from a reactive style (treating emerging problems) to a proactive style of caring (comprehensive assessment of the situation to prevent problems).

*‘There is more structure to it now, while, yeah, 15 years ago, I mean, people did come home to die but without this structure. You were on your own. Palliative care is more like a structured way* [of delivering care] *now, yes. Before, it used to be, yeah, mere symptom control… when something came up, you had to take care of it, you had to treat it.’* (FG1, P1)

Observing palliative-care nurses’ relationships with palliative patients teaches GPs how to deal with complex situations.

*‘You have to experience it in order to learn. That’s something you can see in the relationship between the nurse and the patient. Gee, that’s some relationship! You can learn something from that, how does she* [the nurse] *handle it? You have to see how she goes about it. You can’t write it down. I mean, it is almost a sort of parenting moment.’* (FG5, P1)

This observation was confirmed by the PHCT members:

‘Yeah, a very important thing in this matter is that the learning moment for GPs is mostly situated in the contacts with palliative home-care teams, at the patient’s home. This is the greatest learning moment for most of them.’ (FG3 P3)

In addition to professional growth (acquisition of professional competences), learning through collaboration also seems beneficial to the GPs’ personal growth. A general feeling of safety and trust in the PHCTs enables GPs to discuss their own problems and weaknesses.

*‘I suppose it has something to do with safety, and with relying on experience and expertise and on communication. Not judging or condemning them, like: that doctor seemed to struggle, and now he’s going to share this with us so to speak… because of working with the team nurses for years they dare to admit that they need assistance.’* (FG4, P6)

When considering the composition of a care team for the patient, most GPs were not restrictive and valued the involvement of all caregivers, both professional and non-professional.

In that way, in addition to learning from specialist PHCT nurses, GPs stated they learn a lot from observing the family members’ way of delivering care.

P3:*’I think the conduct of the family is, on the human aspects, sometimes very educational…sometimes everything works out just fine and then you say: well done!’*

P1: *‘You learn mostly how families are functioning’* (FG1)

The GPs’ learning trajectory follows the patient’s actual (on-the-spot) care needs. The patient can even play an active role in stimulating GPs’ learning.

*‘I have a feeling change might come from the patient himself. He’s becoming more empowered, he reads more and he sees more. (He) goes to the GP and says: ‘look, I’ve heard that, I would like that…’ And pushes him to become skilled and experienced in it.’* (FG5, P2)

Palliative-care team participants acknowledge the expressed value of practice-based learning by the GPs and are willing to accept the responsibility of being a facilitator of GPs’ learning.

‘…and that’s one of our positions actually, that we, palliative-care physicians and nurses, are a kind of trainer or coaching team to them.’ (FG4, P2)

Complementary to the bedside learning moments for GPs, learning opportunities are readily available during meetings on the planning of patient care.

‘A care consultation, that hasn’t been installed to educate, but if you want, you can learn a lot on how you would do it and what possibilities you have in your discipline and your organization. I always pass on to my nurses that, if you are invited to a care consultation, first of all for the well-being and continuing care of the patient, you also have to stay alert for learning aspects, and that you can pass things on to the GP at that moment.’ (FG3, P6)

Nevertheless, these meetings are hard to organize since bringing together health-care professionals in primary care is a difficult task.

‘I think that one of the big problems in home care is the fragmentation. I mean, these are all individuals that end up in one and the same situation and they hardly ever meet in person…and maybe we should see how we can link agendas, but I find that practical obstacles can be enormous in a fragmented home situation.’ (FG3, P1)

### What are the views on and preferences for future palliative-care education for GPs according to GPs, CME providers and PHCT members?

a) The need for clinical exposure

As mentioned earlier, the current experiences have led to differing views on the required content of the undergraduate curriculum but there was consensus over the need for clinical experience as part of the education.

All participants believed that the undergraduate curriculum can never be sufficient to prepare a physician for practice, because some aspects of palliative care cannot be learned without clinical experience. Some respondents from palliative-care organisations would like to integrate a palliative-care-unit internship into undergraduate education. Others note the large differences between a palliative-care unit and the home-care situation and fear that this would not be an ideal preparation for GPs.

*‘A couple of days is OK, but it surely isn’t easy, coming from a home-care situation and going to a (palliative care) unit to learn and discover new things. Often, it’s a disillusion when you go back to home care, because of the occasional team set-up and other things you struggle with at home and that work effortlessly in the unit…. I think that’s all valuable indeed but it shouldn’t raise the expectation that it’ll be the same in your work field at home.’* (FG3, P6)

b) Practice-oriented learning

Palliative-care education should mirror palliative-care practice. This has consequences for the content, the format and the organization of CME.

GPs expressed unanimously a strong preference for education on practical issues and concrete advice on how to implement clinical guidelines. Concerning the importance of communication training, however, there was disagreement. Some participants (especially CME providers) stated that repeated and continuous participation in communication training was necessary while others (primarily GPs) doubted this. They stated that only basic training was needed and further skills should be gained through personal experiences.

*’One of the major needs is communication. And communication is something that you don’t learn by going to a lecture. And you don’t learn it by watching videos, but you do learn by practising and training in small groups, and role-play, and with simulated patients.’* (FG3, P5)

*‘Bad news discussion version 36 … you have your basic techniques, and it can beuseful to learn those. But I found my way of applying that technique.’* (FG1, P3)

According to the GPs the best way of delivering CME is by having case-based discussions in small group sessions to see how theories can be put into practice.

*‘Knowledge transfer, and that has been studied, knowledge transfer doesn’t last long. It never changes attitudes. But case-based discussions and peer discussions indeed, those are lasting. And feedback. Doing something and receiving feedback on it.’ *(FG3, P5)

Although CME providers agree with this, they mostly use lecturing as an educational format for CME sessions. They justify this by stating that techniques such as interactive workshops require too much preparation (for which they do not have time), cost too much and require skilled trainers. According to providers of education, teaching is a ‘profession’, and not all good clinicians are good teachers. The art of teaching should be learned during a specific training program.

Some trainers seriously attempted to give this a try but went back to lecturing after having had some disappointing experiences.

*‘I think it’s difficult, you know, outside the palliative care, everyone is giving lectures. In all general courses you can find 90 percent are lectures. The really interactive sessions that took place over the last years…it’s more like a downfall instead of an increase. I sometimes try to get people involved during my talks but it really depends on the group whether it works out or not. Ultimately, case discussions, some will be interesting and some won’t.’* (FG5, P1)

Participants from all groups mentioned the importance of multidisciplinary training but profession-specific courses are required too, since physicians might have a different level of interest in e.g. pharmacology than nurses. General practitioners acknowledge the benefits of professionals from other disciplines (e.g. nurses) acting as trainers/educators. Getting to know each other in this manner facilitates working together as a team afterwards.

‘What’s proven beneficial to learning is putting a group together, I mean putting people from different disciplines together in a shared team to do a training module.’ (FG3, P6)

*‘Well the advantage lies in having a broad view… you get to know other people’s capacities to support you in caring* [for the patient]*.’ (FG3, P3)*

The focus on interprofessional collaborative practice is emphasized by the PHCT members, who state that team working skills are essential for all disciplines.

*‘I think that the poor collaboration between disciplines is something that needs to be put right. Perhaps we should start, in our continuing training, to study with them: how do you work together? And what advantages does it have*.‘ (FG3, P5)

c) Workplace learning conditions

According to the GPs, for collaboration to be effective as a learning moment, there must first of all be readiness to learn.

*’It also depends a lot on your attitude… You have to be open to it, to learn. And not be embarrassed that you don’t know it yet.’ *(FG2, P1)

PHCT members realize that this readiness to learn is not at all self-evident. They see it as an attitude which has to grow gradually, as many GPs are not used to this way of learning.

‘General practitioners often tell me that interprofessional collaboration in a respectful manner is such an important learning moment. They learn from ‘doing things together’. And then returning to it is much easier the next time.’ (FG4, P3)

‘Then there is also the issue of whether these people can effectively open themselves up, through this co-operation, to learn new things, see new elements and new perspectives. Then it is more about an uncertainty and an anxiety about judgments that are going to be shaped rather than an offer that is there and where you have the liberty to use it or not.’ (FG3, P6)

This readiness to learn requires a climate of safety and trust, requiring a careful approach of the learning situation by the PHCT nurses. It may be better to organise a ‘teaching moment’ before or after the bedside encounter and not to display the GPs’ learning need in front of the patient’s family members.

*‘Yes, that was very annoying, the syringe driver was there and then he [the palliative-care nurse]… started to give explanations whilst the whole family was present… he’d better come to our practice beforehand … but then you’re there with the whole family…‘* (FG2, P1)

Although most participants agreed that field training in palliative care (through collaboration with home-care teams) was more effective than attending courses, some PHCT participants report the experience of GPs coming back again and again with their questions because they have forgotten the advice that had been given.

*‘I have a feeling that GPs like bedside training. At least in our team, we see them coming to the team, picking up some items, probably they don’t remember them any longer after one year, and then coming back to the team.’* (FG5, P4)

This was acknowledged by some GPs but others stated that they remembered some information e.g. on practical issues, indicating that workplace learning is not appropriate for all palliative-care content or competences .

‘Practical stuff like using a nasogastric tube or comfort items, yeah, you’ll remember that.’ (FG2, P1)

## Discussion and conclusions

Our study has elicited the experiences and preferences of GPs, PHCT members and CME providers with respect to undergraduate and postgraduate education in palliative care for GPs. Workplace learning has been suggested by participants as a complementary form of lifelong training, with its own specific requirements and conditions.

A first emerging theme is the wish for education to focus on clinical practice, in terms of format as well as content. Upon graduation, GPs do not feel fully prepared to deliver high-quality palliative home care as they lack clear insights in to what palliative care really entails and what will be expected of them in their practice. This reflects the intentions of coordinators of UK medical schools who formulate a concern towards a palliative-care attitude and an awareness of the palliative-care philosophy as an important topic of undergraduate education [25]. The lack of exposure and clinical experience during undergraduate education is mentioned as a major cause of insufficient preparation for practice in our study, confirming the results of a similar study in the UK [26]. The literature describes various ways of introducing practice experience in education with hospice rotation [27-29]. Participants in our study, however, suggest that hospital/hospice training experience cannot easily be transferred to the specific requirements of home-based care. Therefore, it might be interesting to seek for ways of organizing practice rotation in primary care.

Expanding the undergraduate palliative-care curriculum enhances the perception of self-efficacy among students [30]. A valuable alternative, with possibly less impact on the organization of medical schools, might be to analyse the complete medical curriculum for ‘hidden palliative-care content’ (e.g. ‘therapy withdrawal in end-stage cardiac failure’ during lessons in cardiology) and fill in the gaps with a minimum of palliative-care courses [31].

With respect to CME there were clear preferences for interactive, practice-based, small group sessions, thereby confirming literature suggestions on the efficiency of educational formats [32,33]. Unfortunately, as confirmed by a recent review, lecturing remains the primary way of education due to a lack of financial and practical support to provide more interactive training modules [23]. It is worthwhile, however, to make efforts to optimize CME sessions as it has shown the ability to enhance practice [34]. When questioned on the content of CME, GPs preferred it to mirror the complex reality of palliative home care, as is also suggested by the literature [35-37]. With respect to the importance of communication training there was disagreement among the participants. While PHCTs and CME providers call for explicit and repetitive training in communication, GPs prefer to develop their own ways of communication through experience rather than through training sessions. The latter contrasts with the literature, which refers to the positive effects of training on doctors’ communication and promotes interactive training sessions [38,39]. This might be due to the GPs’ reluctance to engage in role-play sessions [40]. The educational outcomes have been shown to be enhanced by practice reinforcement [34].

Workplace learning is the second theme of interest emerging from the results. Practice reinforcement is easily accessible in the case of bedside teaching [41]. This is in line with participants’ preferences for learning by doing. All participants preferred this way of learning to classroom-based learning, especially when addressing GPs’ on-the-spot learning needs. Both GPs (who can be considered the ‘learners’) and PHCT nurses (who can be considered the ‘teachers’, since they are more experienced than the GPs) acknowledge this. The literature on workplace learning (WPL) indicates that this is a reciprocal relationship (both are learning from each other), but the focus of our study was limited to the learning experiences of GPs [42,43]. Participants in our study see WPL as a valuable way of learning, both for practical issues (hands-on training) and for honing a holistic, person-centred attitude towards palliative care (PHCT nurses acting as a ‘role model’). Although the literature supports the idea that a palliative-care attitude should preferably be acquired early in undergraduate medical education in order for GPs to be well-prepared for practice [35,44], participants in our study declare that the PHCT nurses’ role modelling changed their attitude towards palliative patients.

Learning through collaboration offers different ways of learning (e.g. implicit learning, disseminating tacit knowledge) through different learning activities (e.g. observation, receiving feedback) which are difficult to incorporate in CME sessions. Both ways of learning are therefore complementary [17,45]. Opinions on the effectiveness of WPL, however, differ among the participants: while GPs were convinced of the enduring change in competences after a learning experience in a PHCT, the PHCT nurses doubted the effectiveness of it, having witnessed GPs raising the same problems and questions over and over.

A third important theme is the GPs’ self-perception of the tasks and position towards palliative care during interprofessional collaboration. Our study results indicate that palliative care is an integral part of primary care and GPs are willing to make efforts for it, although workload can sometimes limit the GPs’ involvement [14]. Gibbins equally concluded that palliative care is ‘part of being a doctor’ and that the same skills are needed for primary care in general, which is pleaded for in other publications as well [25,46]. PHCTs are a major support for GPs when care becomes too complex. Newly qualified doctors seek support from nurses and the palliative-care team and not from their usual medical team [26,47]. Our study confirms and extends this observation to experienced practicing doctors.

GPs state that they learn from the PHCT nurses through collaboration. Creating opportunities for shared learning and education is a clear indicator that a good partnership between specialist palliative-care services (e.g. PHCTs) and generalists has been established [48]. In our study, the PHCT nurses are willing to take up this responsibility. Professionals positioning themselves as learners, can learn from the more experienced colleagues positioning themselves as learning facilitators [21,49]. As the learner must show a willingness to learn, the facilitator must show a willingness to share knowledge and expertise [50]. The overall concepts of personal identity and professional identity influence the way professionals engage in their work and consequently in workplace learning [51-53]. This means that job perception (the way you define your job and task responsibilities) and self-conception as a practitioner are important [54].

The literature shows that for feedback to be effective, it should be authoritative [55]. Our study shows that authority does not necessarily need to be diploma-based but can also originate from expertise.

An emerging suggestion from some CME providers was to train some interested GPs who can act as an informal reference for their colleagues. In Belgium and other countries there is a lot of experience with formal reference physicians in palliative care who are easily accessible. Some GPs might hesitate to take this ‘official route’ and might prefer to consult a fellow colleague.

Further research is needed to gain insight in the interaction between GPs and PHCT nurses to enhance interprofessional workplace learning.

This study’s greatest strength lies in the integration of the views of all parties involved in palliative home care: GPs, PHCTs and CME providers.

Some limitations have to be mentioned, such as the fact that GPs in Belgium have been used to working with PHCT for many years. This might have influenced their views on learning through collaboration with these teams. Generalizing their views on health-care providers to those from countries without these traditions must be done cautiously. Two different sampling strategies were used: CME providers and PHCT members responded to an invitation to participate in a mail survey, whereas GPs were recruited through a convenience sample of two peer groups. We do not think this has had a major impact on the results since the diversity of participants from CME providers and PHCT members guarantees a broad view on the topic. As for the GPs, since the two groups as a whole agreed to participate, proponents as well as opponents of palliative-care education were present. The predominance of males in the GP groups might have had an influence but reflects the male predominance in the GP workforce (at the time of our study, there were twice as many male GPs in Belgium as female GPs). Fourth, the differing probing questions in the various focus groups might seem to interfere with the analysis of the results but in our view they served to elicit different viewpoints (participants from different backgrounds) on the same topics. The same moderator led all the focus group discussions and ensured that the same topics were discussed in all focus groups. The viewpoint of our study participants may not be representative of the current situation at medical schools (since some participants graduated many years ago) but the expectations they articulate on undergraduate education are probably representative as they were based on current daily work needs (which will always be the patients’ care needs).

In summary, after finishing their undergraduate education, GPs feel unprepared to deliver high-quality palliative care. They also feel insufficiently supported by official CME providers to keep up palliative-care competences. To address their on-the-spot learning needs (induced by specific patient care needs) they turn to PHCTs. While collaborating with these teams, workplace learning occurs. Further research is needed to clarify the dynamics and efficiency of this kind of workplace learning.

## Competing interests

The authors declare that there is no conflict of interest. The authors are solely responsible for the content and writing of this paper.

## Authors’ contributions

PP and SL were involved in the conception and design of the study, the acquisition and analysis of data, the drafting of the manuscript and have given final approval for the version to be published. WJ and VB were involved in the conception and design of the study, the regular revision of the drafts and have given final approval for the version to be published. SA and DM were involved in the interpretation of and discussion on the data, the regular revision of the drafts and have given final approval for the version to be published. All authors read and approved the final manuscript.

## Pre-publication history

The pre-publication history for this paper can be accessed here:

http://www.biomedcentral.com/1471-2296/15/36/prepub
